# Erythrocyte membrane biomimetic EGCG nanoparticles attenuate renal injury induced by diquat through the NF-κB/NLRP3 inflammasome pathway

**DOI:** 10.3389/fphar.2024.1414918

**Published:** 2024-07-09

**Authors:** Jie Qu, Hui Pei, Xin-Ze Li, Yan Li, Jian-Ming Chen, Min Zhang, Zhong-Qiu Lu

**Affiliations:** ^1^ Emergency Department, The First Affiliated Hospital of Wenzhou Medical University, Wenzhou, China; ^2^ Wenzhou Key Laboratory of Emergency and Disaster Medicine, Wenzhou, China

**Keywords:** EGCG, erythrocyte membrane, diquat, NLRP3 inflammasome, oxidative stress

## Abstract

Diquat (DQ) poisoning can cause multiple organ damage, and the kidney is considered to be the main target organ. Increasing evidence shows that alleviating oxidative stress and inflammatory response has promising application prospects. Epigallocatechin gallate (EGCG) has potent antioxidant and anti-inflammatory effects. In this study, red blood cell membrane (RBCm)-camouflaged polylactic-co-glycolic acid (PLGA) nanoparticles (NPs) were synthesized to deliver EGCG (EGCG-RBCm/NPs) for renal injury induced by DQ. Human renal tubular epithelial cells (HK-2 cells) were stimulated with 600 μM DQ for 12 h and mice were intraperitoneally injected with 50 mg/kg b.w. DQ, followed by 20 mg/kg b.w./day EGCG or EGCG-RBCM/NPs for 3 days. The assessment of cellular vitality was carried out using the CCK-8 assay, while the quantification of reactive oxygen species (ROS) was performed through ROS specific probes. Apoptosis analysis was conducted by both flow cytometry and TUNEL staining methods. Pathological changes in renal tissue were observed. The expressions of NLRP3, IL-1β, IL-18, NFκB and Caspase1 were detected by quantitative reverse transcription polymerase chain reaction (qRT-PCR), immunohistochemistry, immunofluorescence, and Western blot. The results showed that the DQ group had increased ROS expression, increased the level of oxidative stress, and increased apoptosis rate compared with the control group. Histopathological analysis of mice in the DQ group showed renal tubular injury and elevated levels of blood urea nitrogen (BUN), serum creatinine (SCr), kidney injury molecule-1 (KIM-1), and cystatin C (Cys C). Furthermore, the DQ group exhibited heightened expression of NLRP3, p-NFκB p65, Caspase1 p20, IL-1β, and IL-18. However, EGCG-RBCm/NPs treatment mitigated DQ-induced increases in ROS, apoptosis, and oxidative stress, as well as renal toxicity and decreases in renal biomarker levels. Meanwhile, the expression of the above proteins were significantly decreased, and the survival rate of mice was ultimately improved, with an effect better than that of the EGCG treatment group. In conclusion, EGCG-RBCm/NPs can improve oxidative stress, inflammation, and apoptosis induced by DQ. This effect is related to the NF-κB/NLRP3 inflammasome pathway. Overall, this study provides a new approach for treating renal injury induced by DQ.

## 1 Introduction

Diquat (DQ), known chemically as 1,1′-ethylidene-2,2′-bipyridine, is a non-selective herbicide commonly found in the form of dibromide. It is a bipyridine compound structurally similar to Paraquat (PQ) (Magalhaes et al., 2018). DQ has gradually become the main herbicide to replace PQ since China issued relevant announcements banning the domestic sales and use of PQ (Xia et al., 2023). DQ poisoning can cause dysfunction in various organs (Burk Rf Fau - Lawrence et al., 1980). Notably, the kidney, as the main excretory organ of DQ, is the main target organ of DQ poisoning (Magalhaes et al., 2018). Current evidence suggests that DQ poisoning patients can occur acute kidney injury (AKI) (Zeng et al., 2023; Yan et al., 2024). In cases of large ingestions, patients can develop symptoms of acute renal failure (Tanen et al., 1999; Fortenberry et al., 2016; Huang et al., 2021). Additionally, DQ can exert toxic effects on other organ systems, including the liver (Burk Rf Fau - Lawrence et al., 1980), lungs (Manabe J Fau - Ogata et al., 1986), heart, central nervous system (Lu et al., 2022), digestive tract, airways (Tanen et al., 1999), skin, and reproductive system (Guangcai et al., 2022; Zhou and Lu, 2022; Yang et al., 2023).

The mechanism of DQ poisoning remains unclear, but a crucial factor is its ability to generate a great deal of reactive oxygen species (ROS) through a series of redox reactions. Upon entering the body, DQ undergoes single-electron addition reactions mediated by NADPH and cytochrome P450 reductase, transforming it into free radicals. These free radicals then react with oxygen (O_2_) to form superoxide anion (O_2_⁻), hydroxyl radicals (·OH), and hydrogen peroxide (H_2_O_2_), among others. This process leads to the excessive production of ROS, causing oxidative stress (Jones and Vale, 2000; Lu et al., 2022). Excessive ROS can directly damage biological macromolecules. Additionally, ROS can act as signaling molecules, activating pathways that lead to the upregulation of inflammatory cytokines (Naik and Dixit, 2011; Abd-Elhakim et al., 2016). Furthermore, hydroxyl radicals can attack the lipid chains of biofilms, initiating lipid peroxidation (Van Vleet and Schnellmann, 2003; Galal et al., 2018). Therefore, research has focused on reducing oxidative stress as a strategy to alleviate organ damage caused by DQ poisoning. Notably, natural compounds derived from Chinese herbs and medicinal plants have been explored as antioxidants for DQ treatment, showing promising therapeutic effects (Chen et al., 2020; Zhang et al., 2020; Jin et al., 2021; Wu et al., 2023).

Epigallocatechin gallate (EGCG), the predominant polyphenolic compound found in green tea, is renowned for its potent antioxidant effects (Shen et al., 2017). Growing evidence suggests that EGCG represents a promising therapeutic or protective agent for various kidney diseases (Kanlaya and Thongboonkerd, 2019). However, the presence of unsaturated bonds in its structure renders EGCG chemically unstable. This instability also leads to low bioavailability, limiting its application in the food and pharmaceutical industries (Liu et al., 2023). Therefore, developing a secure and efficient delivery strategy is crucial to achieving a prolonged release and maintaining the biological activity of EGCG. Nanocarrier delivery systems can improve EGCG’s stability, enable slow release, and even achieve targeted delivery, thereby expanding the potential applications of EGCG (Yan et al., 2020).

Polylactic acid (PLA) and polylactic-co-glycolic acid (PLGA) are widely investigated polymers known for their high biocompatibility (Plucinski et al., 2021). The outer layers of these nanocarriers can be functionalized to specifically target certain cells or structures. These synthetic materials offer high encapsulation rates for EGCG and allow for controlled release (Liu et al., 2023). Several studies have shown that the use of polymeric nanocarriers to deliver EGCG can enhance its therapeutic effect on a variety of diseases. This approach has shown promise in treating cancers, neurodegenerative diseases, and even lessening the occurrence of seizures (Yang et al., 2020; Alrashdi et al., 2023). Furthermore, epigallocatechin gallate Mo nanoparticles (EGM NPs) have been shown to effectively protect against drug-induced hepatic damage (Yang et al., 2022).

However, nanoparticles, being foreign entities, are readily recognized and eliminated by immune system components, hindering their effectiveness (Plucinski et al., 2021). To address this challenge, biomimetic nanoformulations have been developed (Shreffler et al., 2019; Wu et al., 2021). Biomimetic cell membrane nanoparticles leverage natural cell membranes, composed of a cell’s own components, to encapsulate a core made of drugs or simpler nanoparticles (Wang et al., 2019).

Red blood cells have attracted significant interest due to their excellent biocompatibility, easy accessibility, and long lifespan in circulation (Gao et al., 2017). Advancements in cellular and molecular biology have disclosed that red blood cells shield nanoparticle drugs from clearance by the blood and deliver them to targeted organs (Guo et al., 2015). Studies demonstrated the potential of erythrocyte membranes to enhance nanoparticle delivery for treating acute liver failure (Liang et al., 2018). Similarly, studies employed erythrocyte membrane-coated nanoparticles to create a long-circulating sustained-release system for protecting ischemic heart muscle (Huang et al., 2021). Notably, their application in the context of DQ poisoning-induced kidney injury remains unreported.

The NLRP3 inflammasome is the innate immune system’s cornerstone. It recognizes various invading microorganisms and dangerous internal signals through specific pattern-recognition receptors (Sharma and Kanneganti, 2021). Extensive research has shown that the NLRP3 inflammasome-mediated cascade plays a crucial role as an inflammatory signaling pathway, contributing to the progression of various kidney diseases (Bakker et al., 2014; Chi et al., 2017).

Based on the above research background, we hypothesized that PLGA nanoparticles loaded with EGCG and coated with erythrocyte membrane could improve the physicochemical stability of EGCG, thereby improving its bioavailability and therapeutic efficacy. The properties of EGCG-RBCm/NPs were characterized using physical and chemical methods. In addition, we investigated the therapeutic effects and possible mechanisms of EGCG-RBCm/NPs using HK-2 cells and a mouse model of DQ poisoning to explore its potential as a biomimetic nanopreparation for treating kidney injury induced by DQ poisoning.

## 2 Materials and methods

### 2.1 Preparation and characterization of EGCG-RBCm/NPs

PLGA was purchased from Xi’an Ruixi company. RBCm were prepared by differential centrifugation, according to a previous study (Xia et al., 2019). Whole blood (500 μL) was extracted from the orbits of C57BL/6J mice and placed in EDTA-coated tubes, underwent centrifugation at 1,000 rpm for 5 min to eliminate plasma and the white blood cell layer. The residual erythrocytes were then cleansed with chilled PBS to remove serum proteins. Next, they were re-suspended in 25% PBS on ice for 1 h, leading to membrane disruption. Following this, the mixture was spun at 20,000 rpm for 20 min at 4°C, resulting in a pale pink sediment rich in erythrocyte membranes. These membranes underwent sonication (100 W) for 2 min and were sequentially pushed through polycarbonate filters of 400 nm and 200 nm pores using a microextruder. The prepared RBCm were preserved in water at 4°C.

Epigallocatechin gallate nanoparticles (EGCG-NPs) were prepared using the water/oil/water emulsion solvent evaporation method (Wu et al., 2017). 1mg EGCG and 10 mg PLGA were dissolved in dichloromethane for 60 min at room temperature. Then, 0.7 wt% polyvinyl alcohol was added. The obtained solution was treated with an ultrasonic homogenizer for 5 min to obtain EGCG loaded nanoparticles. It was then rinsed three times in distilled water, lyophilized for 48 h, and stored at −80°C. EGCG-RBCm/NPs were synthesized using the extrusion method following a published protocol (Xie et al., 2019). Essentially, a 2 mL suspension of 1 mg/mL EGCG-NPs was amalgamated with RBCm harvested from 500 µL of whole blood, and the blend was agitated magnetically for 45 min. Thereafter, it was sonicated at 100 W for 5 min and passed multiple times through a 200 nm polycarbonate membrane via a microextruder to yield the final EGCG-RBCm/NPs.

Size distribution and zeta potential analysis of PLGA nanocarriers and EGCG-RBCm/NPs were carried out utilizing a dynamic light scattering instrument (Litesizer, Anton Paar, Austria). For a closer examination of the structural appearance of EGCG-RBCm/NPs, a transmission electron microscope (TEM) (JEM-100CX, Japan) was utilized. Moreover, the protein makeup of the EGCG-RBCm/NPs was evaluated through sodium dodecyl sulfate-polyacrylamide gel electrophoresis (SDS-PAGE), followed by staining with Coomassie brilliant blue. The protein bands were visualized using the iBright CL1500 imaging system.

### 2.2 Safety verification of EGCG-RBCm/NPs *in vitro* and *in vivo*

Cell vitality was evaluated through the application of the Cell Counting Kit-8 (CCK-8) assay. Renal tubular epithelial cells of human origin (HK-2) cells were initially cultured in 96-well plates at 37°C in a 5% CO_2_ incubator and allowed to grow for 36 h. Subsequently, the cells were exposed to varying doses of EGCG and EGCG-RBCm/NPs (1–50 μM) for 12 h. After that, each well received a serum-free medium mixed with a 10% CCK-8 reagent and was further incubated for two more hours. Finally, the absorbance at 450 nm was determined using a microplate reader (Tecan Safire, Germany).

Twenty-four C57BL/6J mice were randomly divided into eight groups, with three mice per group. Following a 12-h fast, each group received intraperitoneal (i.p) injections of EGCG and EGCG-RBCm/NPs (20 mg/kg) for 1 day, 2 days, or 3 days, respectively. The mice were then euthanized with 1% sodium pentobarbital, and blood samples, as well as kidney tissues, were collected for subsequent analysis. Blood urea nitrogen (BUN) and serum creatinine (Scr) levels were measured. Kidney tissues were fixed with 4% paraformaldehyde for 24 h, processed through a series of graded alcohol washes to dehydrate them, and sectioned into paraffin sections approximately 5 μm thick. Finally, these sections were stained with H&E for examination of renal tissue morphology under a light microscope (Leica DM750, Germany).

### 2.3 Cell culture

Renal tubular epithelial cells of human origin (HK-2) obtained from the Cell Bank of the Chinese Academy of Sciences were cultured in DMEM-F12 medium (Gibco, United States) supplemented with 10% fetal bovine serum (Gibco) at 37°C in a humidified environment containing 5% CO_2_. To determine the optimal DQ concentration for subsequent experiments, HK-2 cells were exposed to a range of DQ concentrations (1–6,000 μM) for 12 h. Based on cell viability, a concentration of 600 μM DQ was chosen for further studies. Subsequently, HK-2 cells were allocated into the following four groups: 1) Control group: cells were treated with medium for 12 h; 2) DQ group: cells were stimulated with DQ (600 μM) for 12 h; 3) EGCG group: cells were treated with EGCG (15 μM) for 30 min after DQ stimulation, followed by 12 h of incubation.; 4) EGCG-RBCm/NPs group: cells were treated with EGCG-RBCm/NPs (15 μM) for 30 min after DQ stimulation, followed by 12 h of incubation.

### 2.4 Experimental animals

Thirty-six Specific-pathogen-free (SPF) healthy male C57BL/6J mice, aged 6 weeks and weighing 20–25 g, were procured from Zhejiang Weitong Lihua Laboratory Animal Technology Co., Ltd. (Production license number: SCXK (Zhejiang) 2019-0001). Upon arrival, they were acclimatized to a standard laboratory diet for 1 week. The research plan involving animals was approved by the Animal Care and Use Committee of Wenzhou Medical University (IACUC No. Approved:WYYY-IACUC-AEC-2023-049). Prior to the experiment, the mice fasted for 12 h. Using a random number table, the experimental mice were randomly divided into four groups: the control group, the DQ group, the EGCG treatment group, and the EGCG-RBCm/NPs treatment group. Diquat poisoning was induced in the mice through a single intraperitoneal injection of 50 mg/kg DQ (Liang et al., 2007; Xu et al., 2007; Han et al., 2008). The treatment groups received intraperitoneal injections of EGCG or EGCG-RBCm/NPs at a dosage of 20 mg/kg for three consecutive days. Following DQ injection, survival rates and general health of the mice were monitored and recorded every 6 hours for 72 h. After anesthesia induction using 1% sodium pentobarbital, blood was procured through retroorbital bleeding, and renal tissues were collected for subsequent analysis.

### 2.5 Malondialdehyde (MDA) and superoxide dismutase (SOD) assays

The level of lipid oxidation (measured as malondialdehyde) in each group was determined using a chemical colorimetry detection kit (S0131S, Beyotime). Intracellular SOD levels were measured using the WST-8 method with the total SOD activity detection kit (S0101S, Beyotime). The expression levels of MDA and SOD in kidney tissues were assessed using commercially available kits following the manufacturer’s instructions (Nanjing Jiancheng kits: A001-3-2 for MDA, A003-2-2 for SOD).

### 2.6 Determination of reactive oxygen species (ROS)

Intracellular ROS levels in each group were measured using a ROS assay kit (S0033S, Beyotime) with a DCFH-DA fluorescent probe. Fluorescence was then visualized using a laser confocal microscope (Nikon 80i, Japan).

### 2.7 H&E staining

Mouse kidneys were fixed in a 4% paraformaldehyde solution for 24 h, followed by progressive dehydration in ethanol, paraffin embedding, and slicing into 5-micron thick slides. The slides were further processed through defatting, rehydration, and staining with hematoxylin for 10 min and eosin for 1 min using a modified HE staining kit (G1121, Solarbio) according to the provider’s guidelines. After sealing with neutral gum (G8590, Solarbio), renal histomorphological changes were observed under a light microscope (Leica DM750, Germany).

### 2.8 Kidney biomarker detection

SCr and BUN were measured using commercially available kits (C011-2-1, C013-2-1; Nanjing Jiancheng) following the manufacturer’s instructions. ELISA kits (69-21001, 69-30087; MSKBIO) were then used to measure the concentrations of Kidney Injury Molecule-1(KIM-1) and Cystatin C (Cys C) in both serum and kidney tissues.

### 2.9 Flow cytometry (FCM)

Annexin V-FITC apoptosis detection kit (C1062L, Beyotime) was used to detect cell apoptosis. After treatment, cells were harvested, centrifuged at 1,000g for 5 min, and the supernatant was discarded. The cells were then lightly re-suspended with 195 µL Annexin V-FITC binding solution, 5 µL Annexin V-FITC and 10 µL propyl iodide staining solution (Xie et al., 2012; Tang et al., 2015; Zhang et al., 2016). After 15 min of incubation at room temperature in the dark, the samples were analyzed using a BD Aria III flow cytometer (BD Technologies, United States).

### 2.10 TUNEL staining

Apoptosis in the kidney tissue sections was stained using the Terminal Deoxynucleotidyl Transferase (TdT)-Mediated dUTP Nick End Labeling (TUNEL) assay kit (E-CK-A321, Elabscience) according to the supplier’s guidelines. Paraffin sections of kidney tissue were incubated with proteinase K for 20 min. After washing three times with PBS, sections were incubated with 50 μL TdT labeling working solution for 2 h at 37°C in the dark (Tsai et al., 2020; Zhao et al., 2021). The nuclei were counterstained with DAPI (P0131, Beyotime) for visualization. The renal tissue sections were then inspected for apoptotic cells under a fluorescence microscope (Nikon 80i, Japan).

### 2.11 Immunofluorescence

After washing the HK-2 cell slides three times with PBS, the cells were fixed with 4% paraformaldehyde for 15 min at 4°C. They were then permeabilized with 1% Triton for 10 min at room temperature and incubated in a blocking buffer (2% BSA, 0.5% Triton X-100 in PBS) for 1 h. The slides were then incubated overnight at 4°C with diluted primary antibodies against NLR family pyrin domain containing 3 (NLRP3) (1:200), Interleukin-1 beta (IL-1β) (1:200), and Interleukin-18 (IL-18) (1:200). The following day, the slide-mounted cells underwent three washes with PBS before being incubated with Alexa Fluor^®^488-labeled goat anti-rabbit IgG secondary antibody solution (1:1,000, ab150077, Abcam) for an hour at 37°C in darkness. Finally, the samples were sealed with capping tablets containing Hoechst33342. Fluorescence microscopy (Nikon C2si, Japan) was employed to observe the signals of NLRP3, IL-1β, and IL-18. ImageJ software was then used to quantify the positive immunofluorescence signals.

### 2.12 Immunohistochemical analysis

Mouse kidney sections embedded in paraffin were dewaxed and hydrated. To retrieve antigens, these sections were immersed in 0.01M citrate buffer (pH 6.0) and underwent heat-induced antigen retrieval in a microwave oven for 15 min. Subsequently, the sections were treated with 3% H_2_O_2_ at room temperature for 15 min to quench endogenous peroxidase activity, followed by three washes in PBS. Primary antibodies against NLRP3 (1:200), IL-1β (1:200), and IL-18 (1:200) diluted in antibody diluent were applied to the sections and incubated overnight at 4°C. Following overnight incubation, the sections were washed three times with PBS and rewarmed for 20 min on alternate days. After rewarming, the sections underwent three additional washes with PBS, 60 min, the sections were coated with horseradish peroxidase-linked goat anti-rabbit secondary antibody (1:500, A0208, Beyotime). Color development was achieved using a DAB kit (ab64238, Abcam) followed by hematoxylin staining for 10 min. Ultimately, the sections were sealed with neutral resin, and images were captured under a light microscope (Leica DM750, Germany). Positive signals for NLRP3, IL-1β, and IL-18 were quantified using ImageJ software.

### 2.13 qRT-PCR analysis

Total RNA was procured from HK-2 cells utilizing the DP419 Total RNA Extraction Kit (TIANGEN) following the supplier’s protocol. The isolate underwent reverse transcription, converting it into cDNA with the aid of the PrimeScript RT Kit (Thermo Scientific’s RevertAid First Strand cDNA Synthesis Kit). The prepared qPCR system was placed in a PCR instrument and subjected to a denaturation step at 95°C for 1 min, followed by 40 cycles involving 95°C denaturation for 10 s and 60°C annealing for 30 s each. Cq (cycle threshold) values were obtained for each gene (Pei et al., 2024). The relative fold changes in target gene expression were determined by the comparative 2^−ΔΔCt^ (Livak) method using GAPDH as an internal control (Mohamed et al., 2020). The primer sequences detected by Primer Blast are shown in Table 1.

**TABLE 1:** Primer sequences.

Gene	Forward primer (5′ to 3′)	Reverse primer (5′ to 3′)
NLRP3	CCA​GGA​AGA​CAG​CAT​TGA​AGA​GGA​G	GCA​GTC​GTG​TGT​AGC​GTT​TGT​TG
IL-1β	CCG​ACC​ACC​ACT​ACA​GCA​AGG	GGG​CAG​GGA​ACC​AGC​ATC​TC
IL-18	CAT​GCC​CTC​AAT​CCC​AGC​TAC​TC	CTC​GGC​TCA​CCA​CAA​CCT​CTA​C
GAPDH	CAC​CCA​CTC​CTC​CAC​CTT​TGA​C	GTC​CAC​CAC​CCT​GTT​GCT​GTA​G

### 2.14 Western blot analysis

Renal tissue and HK-2 cell lysates were extracted with RIPA buffer (R0010, Solarbio). The protein concentration was assessed by the BCA Protein Assay Kit (P0012, Beyotime). The protein samples were subjected to SDS-PAGE using either 10% or 12% gel and then transferred onto PVDF membranes (IPVH00010, Immobilon®-P) via a wet transfer process. Membranes underwent a 15-min block with a rapid blocking reagent (PS108P, EpiZyme) before being incubated with primary antibodies at 4°C overnight. These antibodies targeted NLRP3 (1:1,000, 27458-1-AP, Proteintech), NFκB p65 (1:1,000, AF5006, Affinity), p-NFκB p65 (1:1,000, AF2006, Affinity), KIM-1 (1:2,000, PA5-20244, Thermo Fisher Scientific), Caspase1 (1:1,000, 22915-1-AP, Proteintech), Caspase1 p20 (1:1,000, AF4005, Affinity), IL-1β (1:1,000, AF5103, Affinity), IL-18 (1:1,000, DF6252, Affinity), and GAPDH (1:5,000, AF7021, Affinity). Following multiple TBST washes, the membranes were treated with HRP-labeled goat anti-rabbit secondary antibody (1:10,000, A0208, Beyotime) for an hour at room temperature. Further TBST washes were conducted, and protein bands were detected by a high-sensitivity ECL chemiluminescence kit (BMU102-CN, Abbkine) and quantitatively analyzed semi-quantitatively using ImageJ software.

### 2.15 Statistical analysis

The analysis of the experimental data was performed using GraphPad Prism 9 software, where outcomes were depicted as the mean ± standard deviation (SD). Each experiment was conducted thrice or more, and statistical evaluation was undertaken utilizing t-tests to assess the mean differences among groups. A *p*-value less than 0.05 was deemed statistically noteworthy. The designations for statistical significance are ^&^ *p* < 0.05, ^&&^ *p* < 0.01, ^&&&^ *p* < 0.005, ^&&&&^ *p* < 0.0001, *p < 0.05, **p < 0.01, ***p < 0.005, ****p < 0.0001, ^#^ *p* < 0.05, ^##^ *p* < 0.01, ^###^ *p* < 0.005, and ^####^ *p* < 0.0001.

## 3 Results

### 3.1 Characterization of EGCG-RBCm/NPs

**FIGURE 1:**
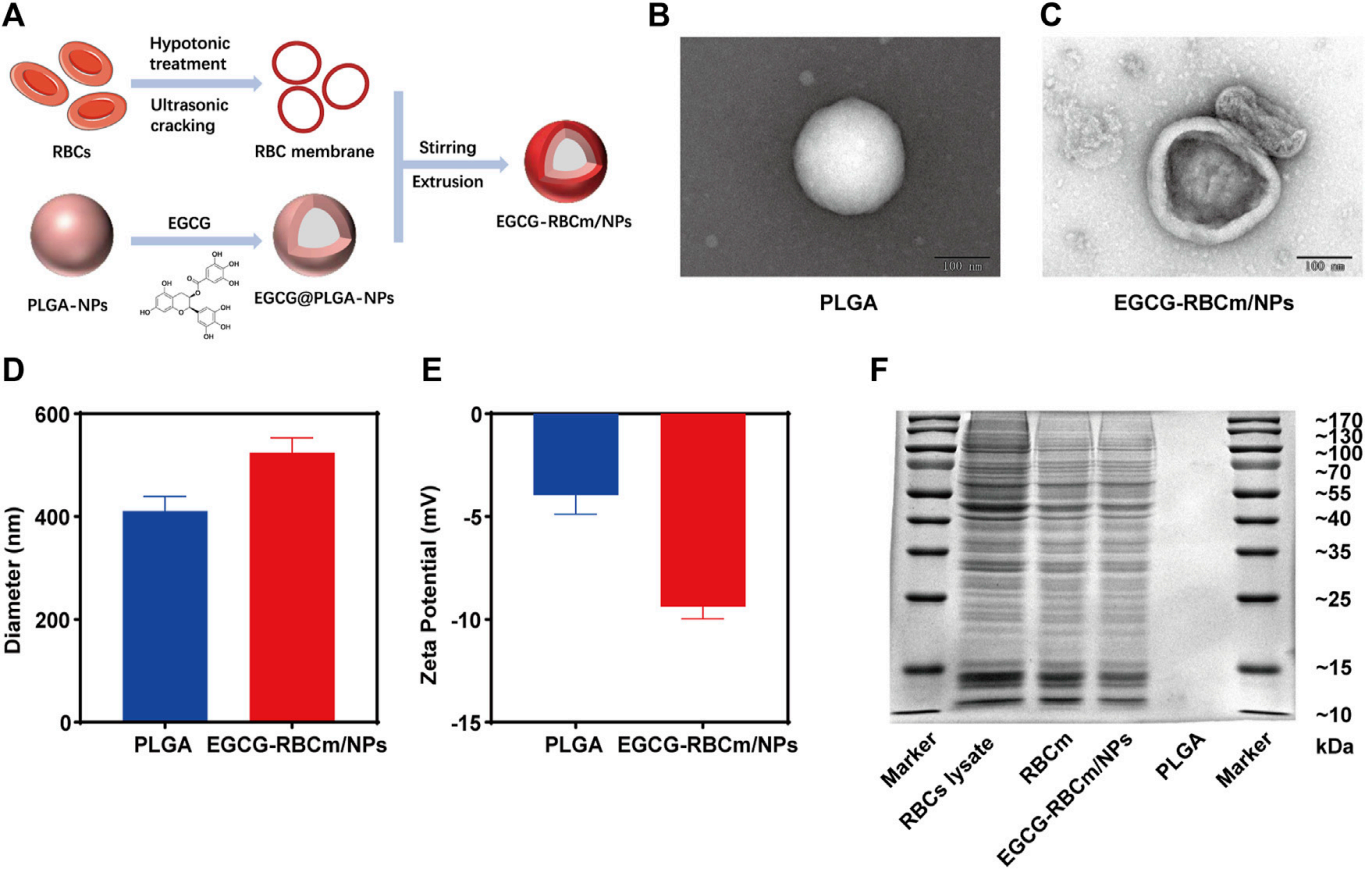
Characterization of red cell membrane bionic EGCG nanoparticles (EGCG-RBCm/NPs). **(A)** Construction of EGCG-RBCm/NPs, **(B, C)** TEM, **(D)** Diameter, and **(E)** zeta potential of PLGA and EGCG RBCm/NPs. **(F)** SDS-PAGE of RBC lysate, RBCm, and EGCG-RBCm/NPs. In **(D, E)**, data are expressed as the mean ± SD (n = 3). Scale bar = 100 nm.

The synthesis of EGCG-RBCm/NPs involved three steps, as illustrated in Figure 1A: extraction of the erythrocyte membrane, preparation of the EGCG-NPs, and coating of the erythrocyte membrane onto the EGCG-NPs. The examination via TEM disclosed that both the PLGA and EGCG-RBCm/NPs demonstrated a consistent spherical shape, characterized by an unblemished, non-porous surface (Figures 1B, C). Notably, EGCG-RBCm/NPs displayed a distinct core-shell nanostructure, with EGCG-NPs encapsulated by the RBCm (Figure 1C). Furthermore, particle size and zeta potential measurements were performed on PLGA and EGCG-RBCm/NPs (Figures 1D, E). The particle size increased from 410.7 ± 28.4 nm for PLGA to 524.3 ± 29.0 nm for EGCG-RBCm/NPs. Correspondingly, the zeta potential shifted from −3.96 ± 0.92 mV (PLGA) to −9.37 ± 0.60 mV for EGCG-RBCm/NPs, which closely resembled the zeta potential of RBCm.

The validation of the RBCm layer on the EGCG-RBCm/NPs was ascertained through conducting a SDS-PAGE gel electrophoresis analysis (Figure 1F). The protein patterns of both RBC lysate/RBCm samples and EGCG-RBCm/NPs nanoparticles were highly similar, suggesting the presence of identical protein components and successful preservation of RBCm on the surface of these nanoparticles.

In conclusion, the above findings substantiate the successful coating of erythrocyte membranes onto EGCG-NPs.

### 3.2 Safety of EGCG and EGCG-RBCm/NPs *in vitro* and *in vivo*

**FIGURE 2:**
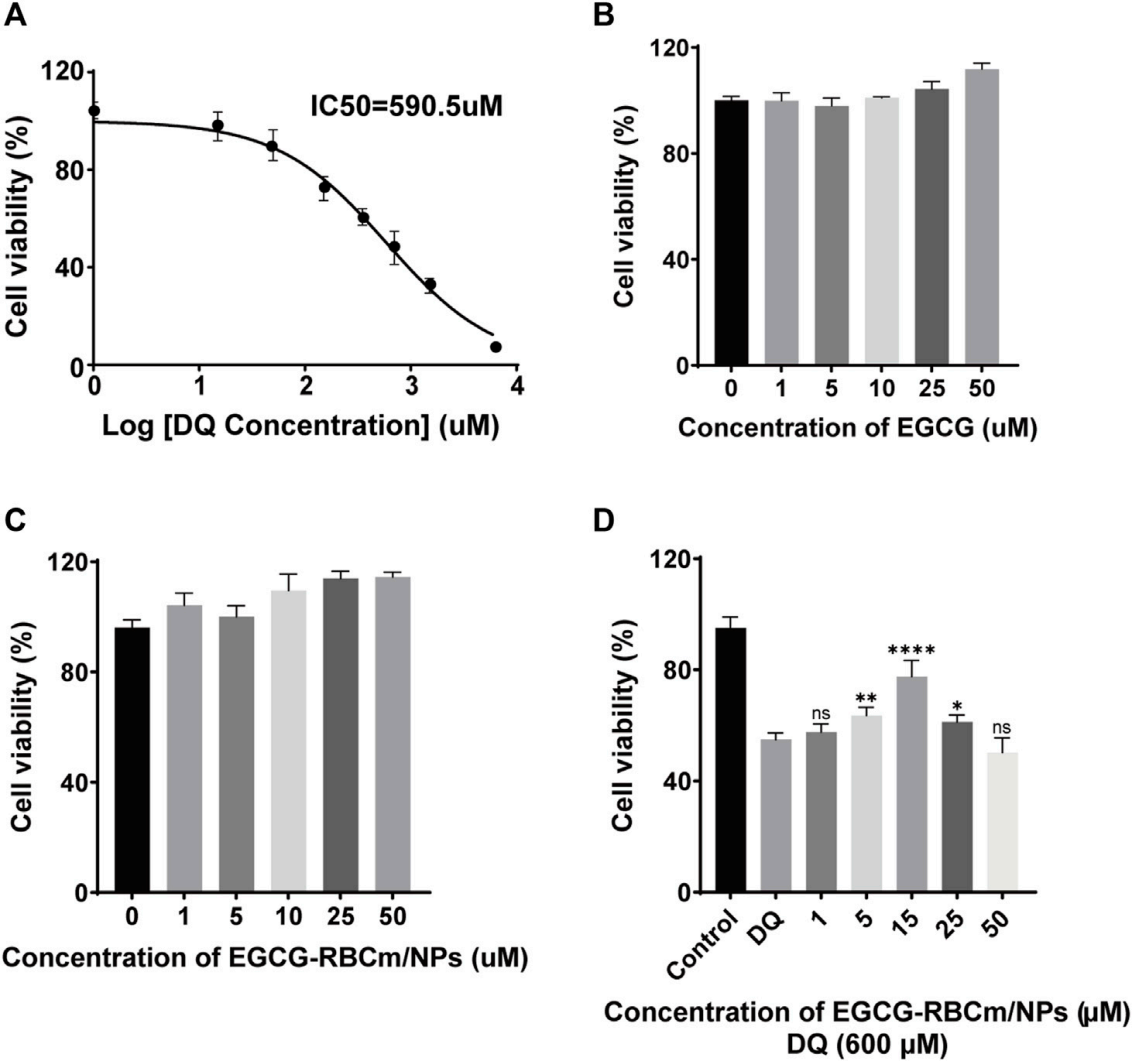
The effects of different concentrations of DQ, EGCG and EGCG-RBCM/NPs on cell proliferation. **(A)** The proliferation of HK-2 cells was detected by CCK8 assay after treatment with DQ (1, 15, 50, 150, 350, 700, 1,400, 3,000, 6,000 μM) for 12 h. **(B)** CCK8 assays were performed to assess the proliferation of HK-2 cells treated with EGCG, **(C)** EGCG-RBCM/NPs (1, 5, 10, 25, 50 μM) for 12 h. **(D)** CCK8 assay was used to assess the proliferation of HK-2 cells co-treated with DQ (600 μM) and EGCG-RBCm/NPs (1, 5, 15, 25, and 50 μM) for 12 h. Data are expressed as the mean ± SD (n = 6). ns *p* > 0.05, *p < 0.05, **p < 0.01, and ****p < 0.0001, vs. DQ group.

**FIGURE 3:**
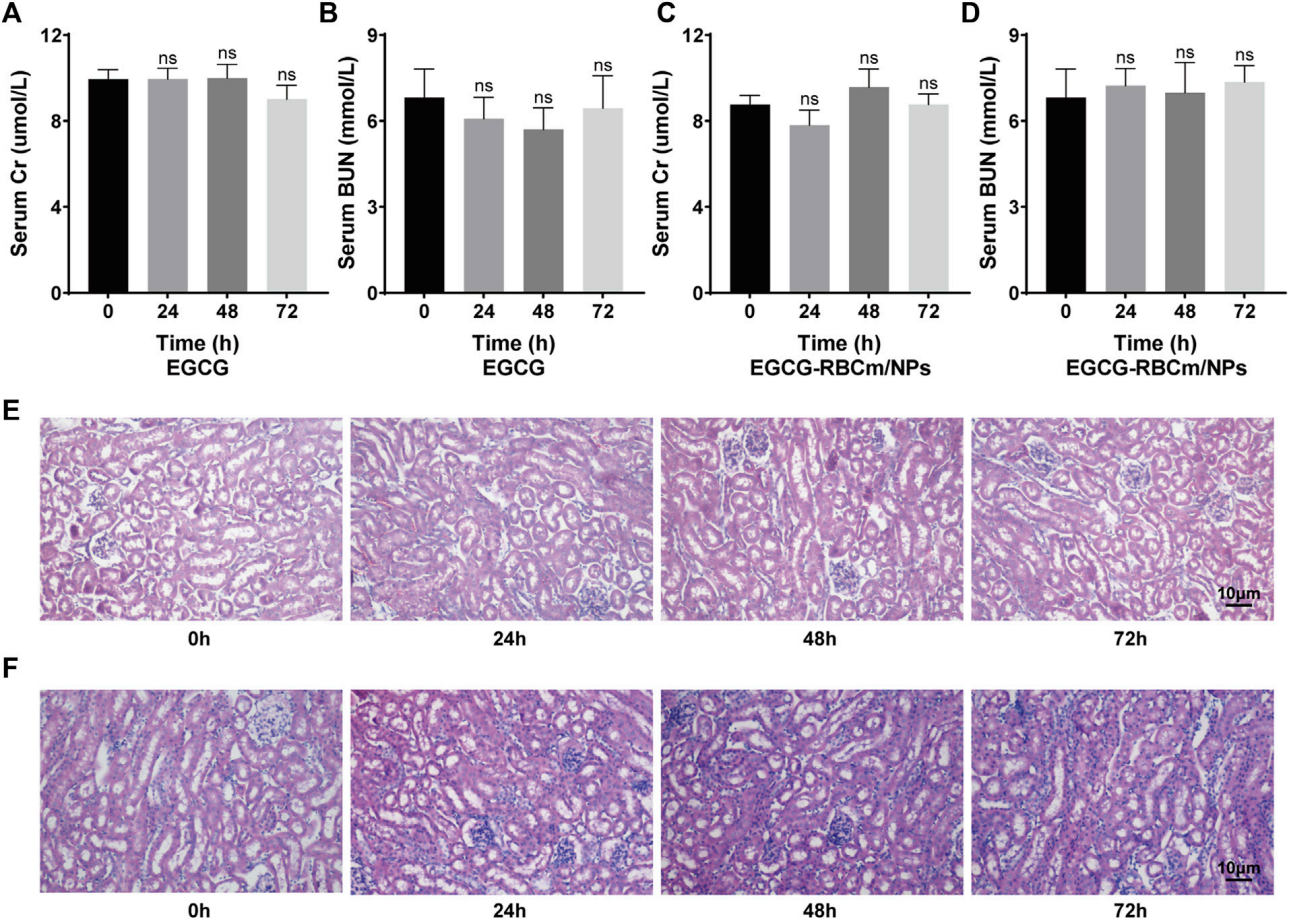
*In vivo* safety evaluation of EGCG and EGCG-RBCM/NPs. **(A, C)** Levels of serum creatinine and **(B, D)** blood urea nitrogen, and **(E, F)** kidney HE staining after continuous administration of EGCG or EGCG-RBCM/NPs for 24 h, 48 h, and 72 h, respectively. Data are expressed as the mean ± SD (n = 3). Scale bar = 10 μm.

Treatment with different concentrations of EGCG or EGCG-RBCm/NPs did not significantly alter HK-2 cell viability across the range of 1–50 μM (Figures 2B, C). This suggests that EGCG-RBCm/NPs exhibited lower cytotoxicity and may have a proliferative effect on HK-2 cells compared to EGCG alone. Intraperitoneal injection of EGCG or EGCG-RBCm/NPs did not cause significant changes in SCr or BUN levels contrasted with the control group (Figures 3A–D). Histopathological analysis of kidney tissue sections using H&E staining revealed no apparent abnormalities in renal tubules, glomeruli, or other structures (Figures 3E, F). These findings suggest that both EGCG and EGCG-RBCm/NPs have minimal negative effects on kidney function and histology.

### 3.3 The therapeutic effect of EGCG-RBCm/NPs on DQ-induced HK2 cell injury

**FIGURE 4:**
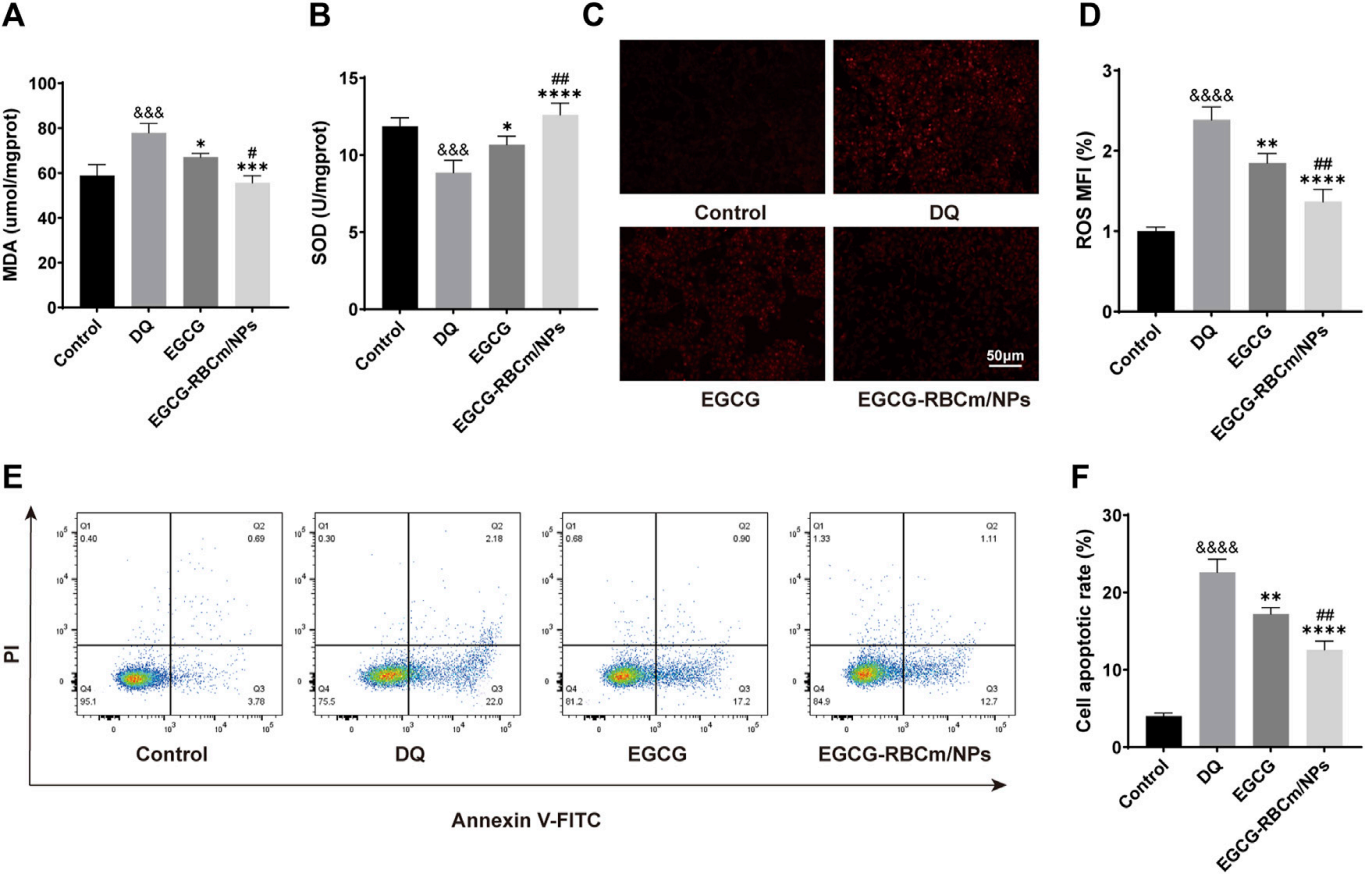
*In vitro* antioxidant and anti-apoptotic efficacy of EGCG-RBCm/NPs. HK-2 cells stimulated with DQ (600 μM) were treated with EGCG and EGCG-RBCM/NPs (15 μM). MDA **(A)** level and SOD **(B)** activity in each group of cells. **(C)** Intracellular reactive oxygen species (ROS) in each group. **(D)** Semi-quantitative analysis of the mean fluorescence intensity (MFI) of ROS. Apoptosis was assessed by **(E)** flow cytometry and **(F)** quantitative analysis. Data are expressed as the mean ± SD (n = 3). Scale bar = 50 μm ^&&&^ *p* < 0.005, ^&&&&^ *p* < 0.0001, vs. Control group; *p < 0.05, **p < 0.01, ***p < 0.005, and ****p < 0.0001, vs. DQ group; ^#^ *p* < 0.05, ^##^ *p* < 0.01, vs. EGCG group.

DQ stimulation resulted in a dose-dependent decrease in HK-2cell viability (Figure 2A). EGCG-RBCm/NPs demonstrated the most effective ability to mitigate DQ toxicity at a concentration of 15 μM (Figure 2D). Compared to the control group, cellular MDA content increased while SOD activity decreased following DQ stimulation. However, treatment with EGCG-RBCm/NPs significantly reversed these trends. MDA content was decreased by 28.69% (*p* < 0.005) and SOD activity was increased by 42.18% (*p* < 0.005) compared to the DQ-treated group (Figures 4A, B). Notably, the therapeutic effect of EGCG-RBCm/NPs was superior to that of EGCG alone, demonstrating significant differences (*p* < 0.05, *p* < 0.005). Measurement of intracellular ROS using a fluorescent probe revealed a significant increase in their levels within HK-2 cells after DQ stimulation. Nevertheless, treatment with EGCG-RBCm/NPs reduced ROS fluorescence intensity by 42.73% (*p* < 0.0001) (Figures 4C, D). Unstimulated HK-2 cells exhibited an apoptosis rate of roughly 4%, as revealed by Annexin V/PI-based flow cytometric assessment of apoptosis. This rate rose to 22% after DQ stimulation but significantly dropped to around 12.7% following treatment with EGCG-RBCm/NPs (Figures 4E, F).

Collectively, these data demonstrate that EGCG-RBCm/NPs effectively protect HK-2 cells from DQ-induced injury by diminishing ROS generation, inhibiting apoptosis, and mitigating oxidative stress.

### 3.4 The therapeutic effect of EGCG-RBCm/NPs on DQ-induced acute kidney injury

**FIGURE 5:**
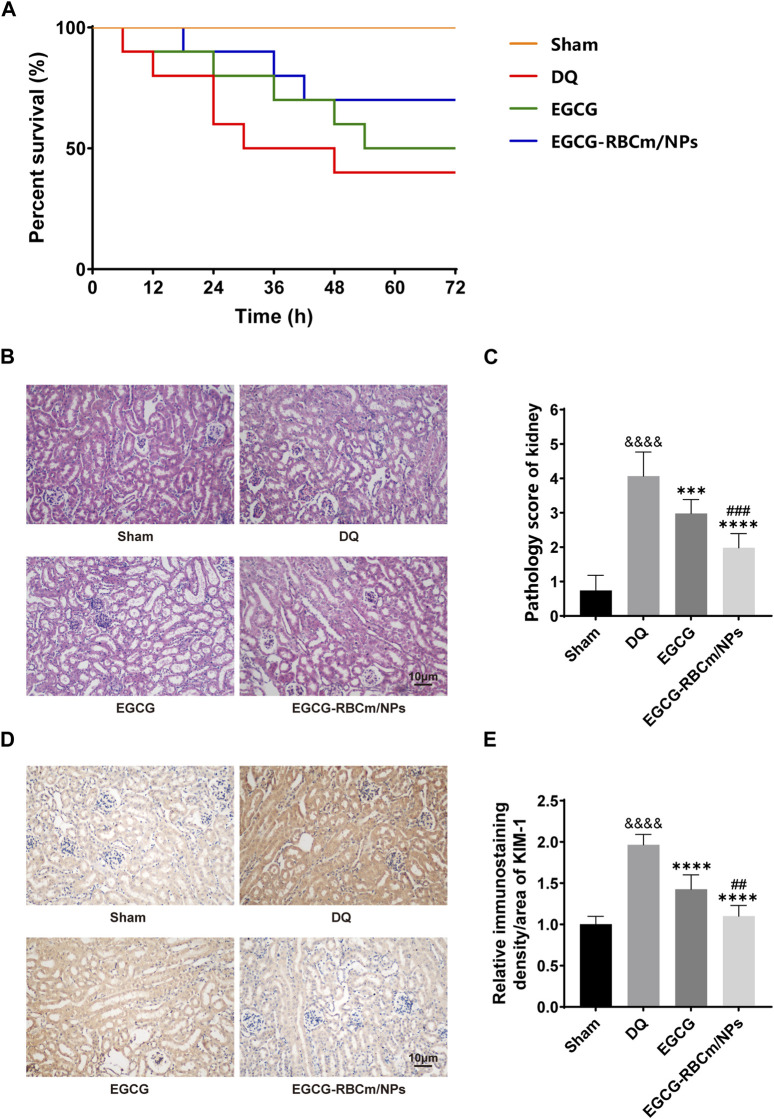
Protective effects of EGCG-RBCm/NPs on DQ-poisoned mice. **(A)** Kaplan-Meier survival curves. **(B)** Renal injury score, **(D)** HE staining of mouse kidney tissues. **(E)** Mouse kidney KIM-1 immunohistochemistry, and **(C)** semi-quantitative analysis. Data are expressed as the mean ± SD (n = 5). Scale bar = 10 μm ^&&&&^ *p* < 0.0001, vs. Control group; ***p < 0.005, ****p < 0.0001, vs. DQ group; ^##^ *p* < 0.01, ^###^ *p* < 0.005, vs. EGCG group.

Following DQ intoxication, mice were treated with either EGCG or EGCG-RBCm/NPs to evaluate their therapeutic efficacy. The survival rate and overall health of the mice in each group were monitored. The mortality and general health status of mice in each group were monitored. As depicted in Figure 5A, at 72 h, the mortality of mice in EGCG-RBCm/NPs group was 30%, DQ group was 60%, and EGCG group was 50% (*p* < 0.05). Mice in the DQ group displayed a deteriorated condition compared to the treatment groups, characterized by alopecia, reduced locomotor activity, cognitive impairment, and convulsions. These results suggest that EGCG-RBCm/NPs significantly improved the survival rate and health state of mice with DQ poisoning.

**FIGURE 6:**
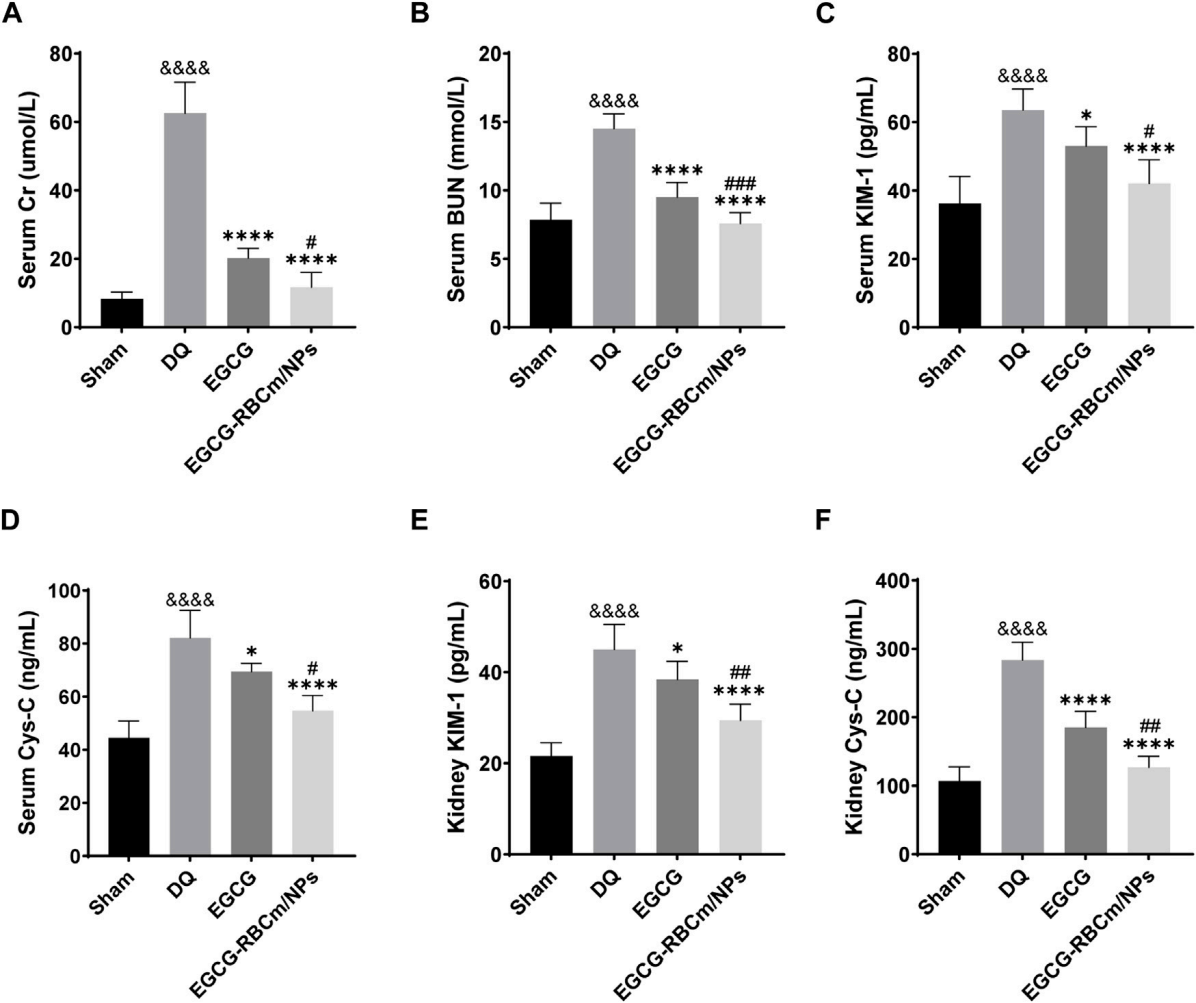
EGCG-RBCm/NPs reduced the levels of renal injury markers in DQ-poisoned mice. Levels of serum creatinine **(A)** and blood urea nitrogen **(B)** in mice. Serum KIM-1 **(C)** and Cys-C **(D)** levels in mice. Kidney KIM-1 **(E)** and Cys-C **(F)** levels in mice. Data are expressed as the mean ± SD (n = 5). ^&&&&^ *p* < 0.0001, vs. Control group; *p < 0.05, ****p < 0.0001, vs. DQ group; ^#^ *p* < 0.05, ^##^ *p* < 0.01 and ^###^ *p* < 0.005, vs. EGCG group.

H&E staining of kidney tissue sections from the control group revealed normal morphology with a regular arrangement of cells, intact tubular structures, and minimal infiltration of inflammatory cells. In contrast, the DQ group displayed swollen and disorganized renal tubular epithelial cells, disruption of tubular architecture, cellular debris, casts, and infiltration of inflammatory cells. However, treatment with EGCG-RBCm/NPs significantly improved these pathological changes observed in the kidneys (Figures 5B, C). Moreover, the application of DQ led to a marked upsurge in SCr and BUN levels when contrasted with the untreated group. Conversely, treatment with EGCG-RBCm/NPs reduced SCr by 81.33% and BUN by 47.76% compared to the DQ-treated group (*p* < 0.0001) (Figures 6A, B), exhibiting a more robust therapeutic outcome than EGCG by itself (*p* < 0.05, *p* < 0.005). To delve deeper into the renal protective influence of EGCG-RBCm/NPs, KIM-1 and Cys C levels were quantified in both serum and kidney tissues using ELISA kits. DQ exposure caused a substantial hike in KIM-1 and Cys C levels in both serum and kidney tissue compared to the control (*p* < 0.0001). In contrast, treatment with EGCG-RBCm/NPs decreased KIM-1 by 33.73% and 33.46%, Cys C by 33.39% and 55.22% (*p* < 0.05) (Figures 6C–F). Supporting these findings, immunohistochemical staining with a KIM-1 antibody revealed heightened KIM-1 expression in the DQ group, whereas it was decreased by 44.03% in the EGCG-RBCm/NPs group (Figures 5D, E).

**FIGURE 7:**
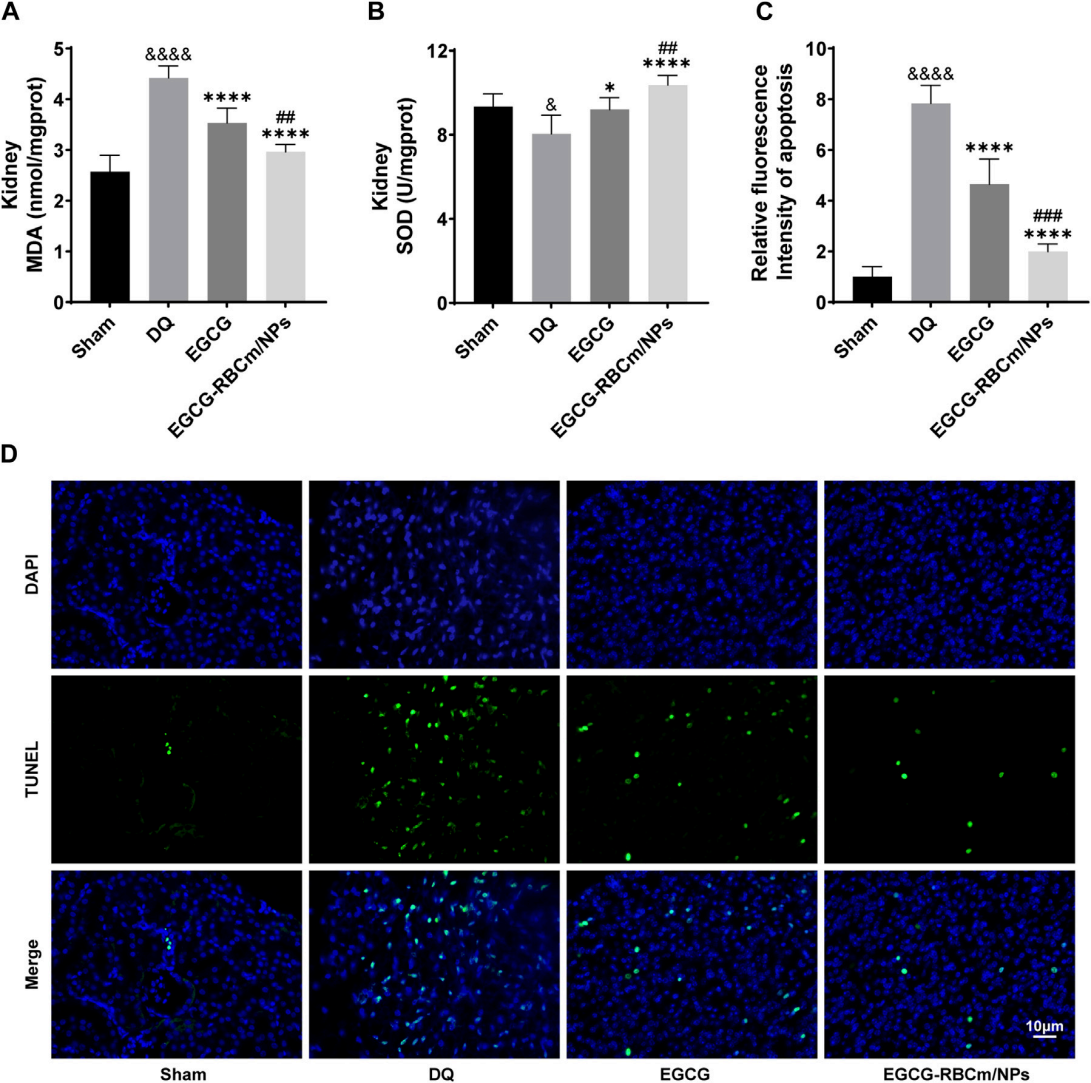
EGCG-RBCm/NPs significantly inhibited oxidative stress and cell apoptosis in DQ-poisoned mice. **(A)** MDA level in kidney tissue of mice; **(B)** SOD activity in kidney tissue of mice. **(D)** Representative images of TUNEL staining (green) and DAPI staining (blue) and semi-quantitative analysis **(C)**. Data are expressed as the mean ± SD (n = 5). Scale bar = 10 μm ^&^ *p* < 0.05, ^&&&&^ *p* < 0.0001, vs. Control group; *p < 0.05, ****p < 0.0001, vs. DQ group; ^##^ *p* < 0.01, ^###^ *p* < 0.005, vs. EGCG group.

Kidney tissues from DQ-treated mice displayed significantly higher MDA content and lower SOD activity contrasted with the control group. Nevertheless, treatment with EGCG-RBCm/NPs reduced MDA content by 32.97% (*p* < 0.0001) and increased SOD activity by 28.81% (*p* < 0.0001) (Figures 7A, B). TUNEL staining revealed significantly increased cell apoptosis and DNA damage in kidney tissues from the DQ group compared to the control group. Notably, EGCG-RBCm/NPs treatment reduced apoptosis cells by 74.48% compared to the DQ group (*p* < 0.0001) (Figures 7C, D). While EGCG yielded therapeutic effects, it was significantly less pronounced than that observed with EGCG-RBCm/NPs (*p* < 0.0005). These findings collectively indicate that EGCG-RBCm/NPs exert therapeutic effects against DQ-induced AKI by suppressing oxidative stress and attenuating renal apoptosis caused by DQ exposure.

### 3.5 EGCG-RBCm/NPs inhibited the activation of the NLRP 3 inflammasome pathway and NF-κB in DQ-poisoned mice

**FIGURE 8:**
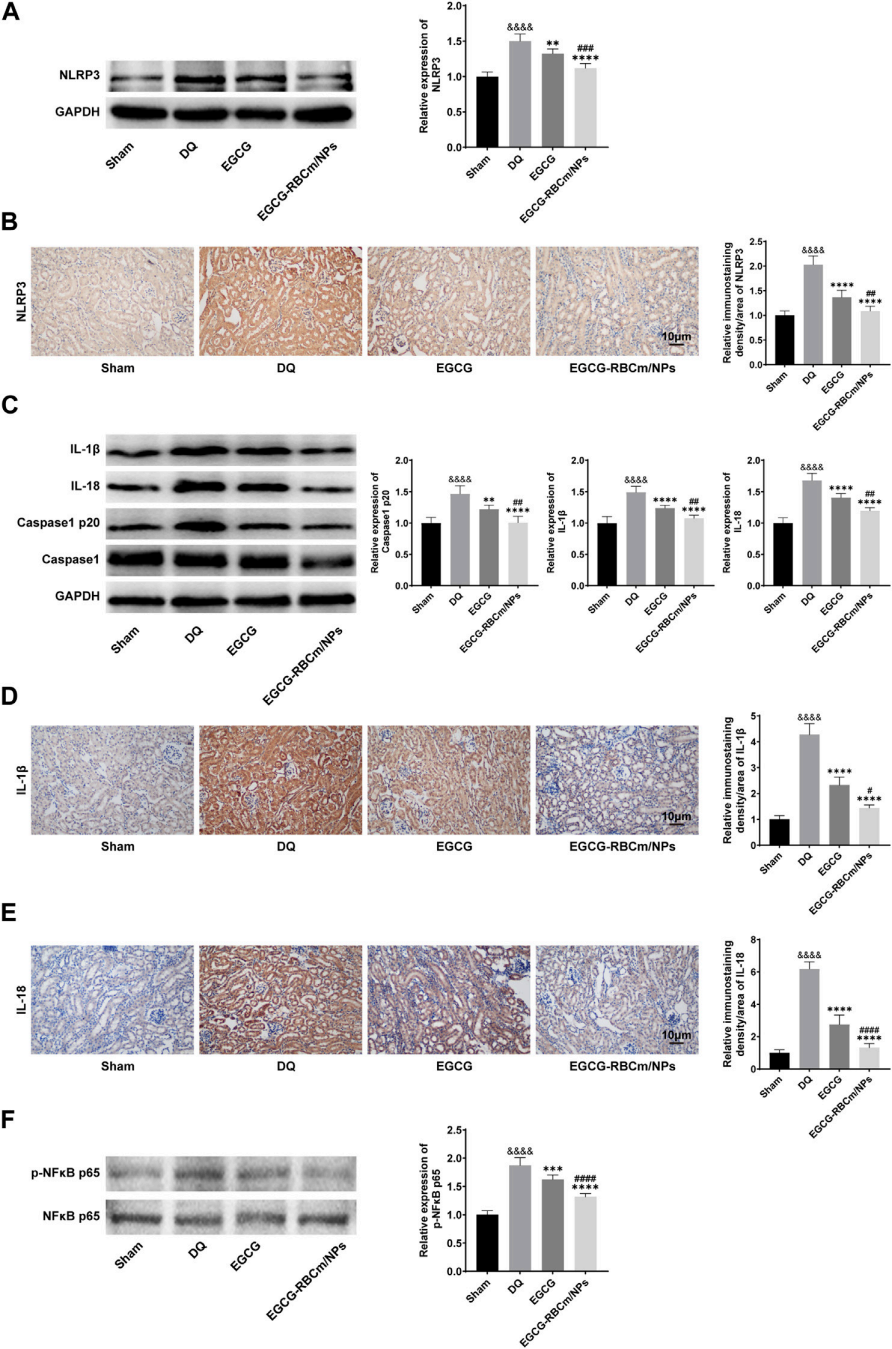
The effects of EGCG-RBCm/NPs on NF-κB and NLRP 3 inflammasomes in DQ-poisoned mice. After treatment with EGCG or EGCG-RBCM/NPs (20 mg/kg) and DQ (50 mg/kg), the protein levels of NLRP 3 **(A)**, IL-1β, IL-18, Caspase1, Caspase1 p20 **(C)**, p-NFκB p65 and NFκB p65 **(F)** in kidney tissue were detected by Western blot. Immunohistochemical and semi-quantitative analysis of NLRP 3 **(B)**, IL-1β **(D)**, and IL-18 **(E)**. Data are expressed as the mean ± SD (n = 5). Scale bar = 10 μm ^&&&&^ *p* < 0.0001, vs. Control group; *p < 0.05, ***p < 0.005 and ****p < 0.0001, vs. DQ group; ^#^ *p* < 0.05, ^##^ *p* < 0.01, ^###^ *p* < 0.005 and ^####^ *p* < 0.0001, vs. EGCG group.

To explore the fundamental mechanism governing the function of EGCG-RBCm/NPs, we analyzed their effect on the NLRP3 inflammasome pathway through protein expression analysis via Western blot in kidney tissue lysates from different groups of mice. The NLRP3 inflammasome pathway is a key component of the immune system that can contribute to inflammatory responses in various diseases, including kidney injury. By suppressing NLRP3 inflammasome activation, EGCG-RBCm/NPs may offer a protective mechanism against DQ-induced kidney damage. The findings revealed that compared to the control group, NLRP3 protein expression was increased by 50.1% in DQ group but decreased by 25.58% in the EGCG-RBCm/NPs group compared to the DQ group (*p* < 0.0001) (Figure 8A). Additionally, Caspase1 p20, IL-1β, and IL-18 protein expression were increased by 46.80%, 49.20% and 68.00% in DQ group mice compared to the control group but decreased by 31.34%, 27.88%, and 28.81% in the EGCG-RBCm/NPs group compared to the DQ group (*p* < 0.0001) (Figure 8C). Furthermore, immunohistochemical staining of kidney tissues confirmed these findings (Figures 8B, D, E). The protein expressions of NLRP3, IL-1β and IL-18 in the EGCG-RBCm/NPs group were decreased by 46.35%, 66.38% and 78.55% compared to the DQ group (*p* < 0.0001). In conclusion, these findings suggest that EGCG-RBCm/NPs can inhibit activation of the NLRP3 inflammasome pathway, consequently reducing renal damage caused by DQ.

To investigate the function of NF-κB in NLRP3 inflammasome activation in DQ-triggered mice, we examined protein expression levels of NFκB p65 and its phosphorylated form (p-NFκB p65). The Western blot analysis revealed an elevation in p-NFκB p65 expression in DQ-induced mice compared to the control group, while the expression of NFκB p65 protein remained unchanged. EGCG-RBCm/NPs treatment reduced the expression of p-NFκB p65 by 29.71% compared to the DQ group. Notably, EGCG-RBCm/NPs exhibited a more pronounced suppressive effect, with statistically significant differences compared to the control group (*p* < 0.0001) and the EGCG group (*p* < 0.01, *p* < 0.0005) (Figure 8F).

### 3.6 EGCG-RBCm/NPs inhibited the activation of the NLRP3 inflammasome pathway and NF-κB in HK-2 cells

**FIGURE 9:**
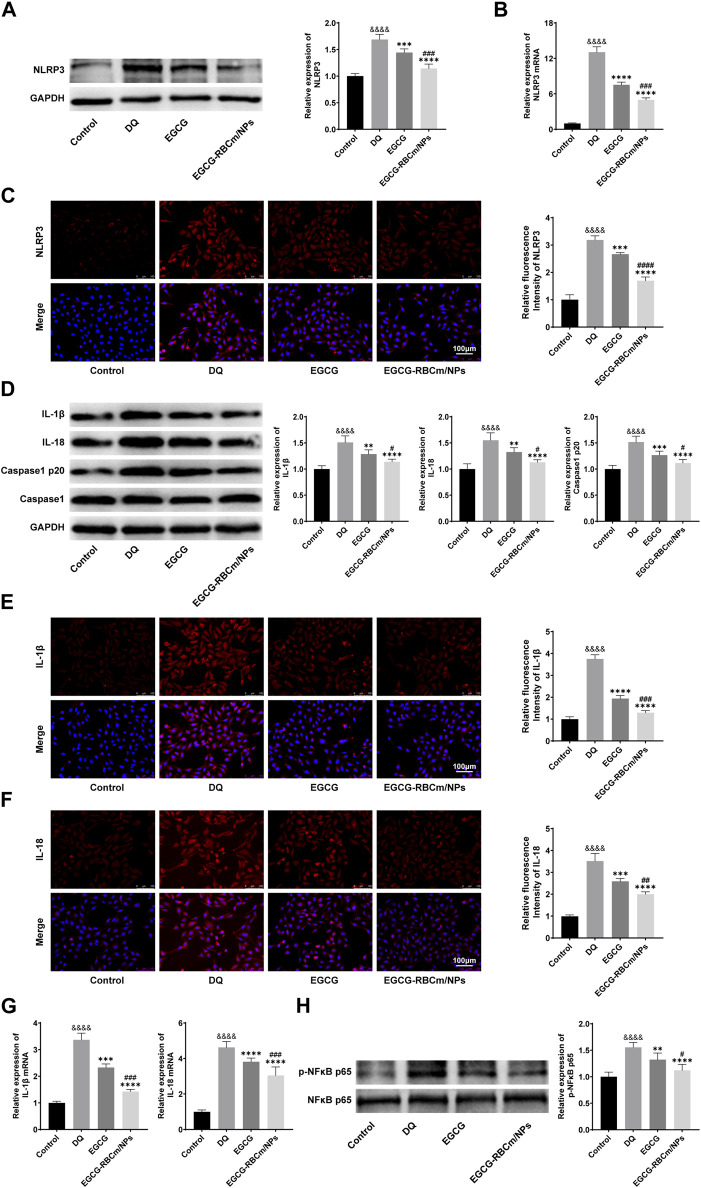
The effects of GCG-RBCm/NPs on NF-κB and NLRP 3 inflammasome in DQ-induced HK-2 cells. Western blot analysis of EGCG or EGCG-RBCM/NPs (15 μM) and DQ (600 μM) for 12 h. Changes in the protein levels of NLRP 3 **(A)**, IL-1β, IL-18, Caspase1, Caspase1 p20 **(D)**, p-NFκB p65, and NFκB p65 **(H)** in HK-2 cells. Immunofluorescence staining and semi-quantitative analysis of NLRP 3 **(C)**, IL-1β **(E)** and IL-18 **(F)**. Changes in NLRP 3 **(B)**, IL-1β, and IL-18 **(G)** mRNA levels were analyzed by qRT-PCR. Data are expressed as the mean ± SD (n = 3). Scale bar = 100 μm ^&&&&^ *p* < 0.0001, vs. Control group; **p < 0.01, ***p < 0.005 and ****p < 0.0001, vs. DQ group; ^#^ *p* < 0.05, ^##^ *p* < 0.01, ^###^ *p* < 0.005 and ^####^ *p* < 0.0001, vs. EGCG group.

To investigate NLRP3 inflammasome activation in HK-2 cells and the protective effects of EGCG-RBCm/NPs, we examined the expression of key proteins. Western blot analysis, qRT-PCR, and immunofluorescence staining revealed a significant increase in NLRP3 inflammasome components in the DQ group compared to the control group (*p* < 0.0001) (Figure 9). Notably, EGCG-RBCm/NPs significantly inhibited this upregulation of NLRP3 (*p* < 0.0001), NLRP3 mRNA expression was decreased by 61.82%, NLRP3 fluorescence intensity was decreased by 46.89%, and NLRP3 protein expression was decreased by 32.13%, and a more pronounced effect compared to the EGCG group (*p* < 0.005) (Figures 9A–C).

We further assessed the expression of Caspase-1 p20, IL-1β, and IL-18, which are downstream effectors of the NLRP3 inflammasome pathway. Western blot analysis showed increased protein levels of these molecules following DQ stimulation, but treatment with EGCG-RBCm/NPs reduced the protein expression by 26.40%, 24.52%, and 27.21%, respectively (*p* < 0.05, *p* < 0.0001) (Figure 9D). Similarly, qRT-PCR demonstrated elevated mRNA levels for IL-1β and IL-18 in the DQ group compared to the control group. Immunofluorescence staining confirmed these findings, revealing increased positive expression in the DQ group. However, compared to the DQ group, treatment with EGCG-RBCm/NPs reduced IL-1β and IL-18 mRNA levels by 57.66% and 34.20% (Figure 9G), and fluorescence intensity by 57.66% and 34.20% (Figures 9E, F), respectively (*p* < 0.05, *p* < 0.005, *p* < 0.0001). In conclusion, these *in vitro* studies strongly suggest that EGCG-RBCm/NPs exert an inhibitory effect on NLRP3 inflammasome activation, which likely contributes to their protective effects against DQ-induced injury in HK-2 cells.

Next, we sought to assess whether the NF-κB pathway and NLRP3 inflammasome are activated simultaneously in HK-2 cells. Accordingly, we examined the phosphorylation level of NF-κB, a marker of its activation. Western blot analysis showed that p-NFκB p65 expression was increased by 55.6% in DQ-stimulated HK-2 cells compared to the control group (*p* < 0.0001) (Figure 9H). Importantly, treatment with EGCG-RBCm/NPs reduced p-NFκB p65 by 27.89% compared to the DQ group (*p* < 0.0001) (Figure 9H).

These findings suggest that the NF-κB pathway might play a role in regulating NLRP3 inflammasome activation during HK-2 cell injury. Importantly, EGCG-RBCm/NPs attenuate this process, potentially contributing to their protective effects.

## 4 Discussion

Diqaut (DQ), a fast-acting, non-selective herbicide, is rapidly absorbed after oral ingestion and is distributed throughout the body, leading to widespread tissue and organ toxicity (Meng et al., 2022). Following oral ingestion and absorption, this herbicide quickly distributes throughout the body, particularly targeting the kidneys, which are highly vulnerable to DQ poisoning (McCarthy et al., 1983). This vulnerability can lead to acute tubular necrosis and renal failure (Cui et al., 2023; Yan et al., 2024). Currently, it is believed that the main toxicological mechanism of DQ poisoning involves the generation of free radicals, likely superoxide radicals, through the reduction-oxidation cycle in cells, resulting in oxidative stress-induced cell dysfunction and subsequent organ impairment (Van Vleet and Schnellmann, 2003; Xu et al., 2007). Despite advancements in organ function support technology, DQ poisoning still carries a high mortality rate, posing an urgent challenge in the medical field.

EGCG, the main component of green tea, possesses potent antioxidant, anti-inflammatory, and anti-apoptotic effects (Huang et al., 2020; Chiu et al., 2021). EGCG has demonstrated pharmacological potential in treating various diseases, including cancer, diabetes, nervous system disorders, cardiovascular diseases, and kidney diseases (Bao et al., 2015; Kanlaya and Thongboonkerd, 2019; Zhao et al., 2019; Aggarwal et al., 2022; Mohd Sabri et al., 2022). For example, a study by Shen et al. (2017) showed that EGCG protected mice from DQ-induced acute lung injury by inhibiting toll-like receptor (TLR) and adapter protein upregulation and NF-κB activation in lung cells. Similarly, Tsai et al. (2011) reported that EGCG could prevent lupus nephritis development and inhibit renal NLR family pyrin domain containing 3 (NLRP3) inflammasome activity through activation of the Nrf2 antioxidant pathway to protect kidney health. However, its limited bioavailability and stability hinder its clinical application (Yang et al., 2020).

Therefore, in this study, we developed erythrocyte membrane PLGA nanoparticles loaded with EGCG (EGCG-RBCm/NPs) as a biomimetic nanomedicine to enhance EGCG stability and investigate its impact on DQ-induced renal injury. The results demonstrated that the administration of EGCG-RBCm/NPs exhibited excellent biocompatibility and significantly ameliorated renal function and histopathological changes in mice, consistent with previous studies (Chang et al., 2022; Sudha et al., 2022).

The pathogenesis of DQ poisoning involves multiple pathways, with current understanding suggesting that multi-organ damage stems from excessive ROS production, leading to an imbalance between antioxidant defenses and oxidative stress (Valavanidis et al., 2006). Excess ROS results from DQ poisoning surpasses the body’s self-clearance ability, activating inflammatory signaling pathways in cells, further exacerbating oxidative stress, and causing cell and tissue damage (Zhang et al., 2022). Our study detected increased levels of ROS in the DQ group, which were inhibited by EGCG-RBCm/NPs. Consistent with the increased ROS, the DQ group displayed elevated MDA, a marker of lipid peroxidation, and decreased SOD, an antioxidant enzyme, indicating heightened oxidative stress (Abdel Fattah et al., 2008). Treatment with EGCG-RBCm/NPs reversed these changes, supporting its antioxidant capacity. Apoptosis, a programmed cell death process, can also arise from oxidative stress and play a role in tissue injury. Research has revealed that DQ can induce apoptosis in various cell types (Wu et al., 2012). Aeri Park et al. discovered that NF-κB and p53-dependent apoptosis and autophagy are involved in DQ-induced neurotoxicity in PC12 cells (Park and Koh, 2019); however, antioxidants can alleviate this toxicity, suggesting that autophagy and apoptosis also contribute to DQ pathogenesis. Our study confirmed that DQ induces apoptosis in kidney cells both *in vitro* and *in vivo*; nevertheless, treatment with EGCG-RBCm/NPs reduced the rate of apoptosis. These results align with previous studies reporting the antioxidant and anti-apoptotic effects of EGCG (Gao et al., 2016; Rasheed et al., 2017; Liang et al., 2019).

Growing evidence indicates that renal inflammation holds a pivotal role in the development of numerous kidney disorders. This inflammatory response is primarily driven by cellular dysfunction involving pattern recognition receptors (PRRs), particularly nucleotide-binding oligomerization domain receptors (NLRs). NLRs, as intracellularly expressed PRRs, are indispensable for both innate immune reaction and maintaining tissue equilibrium (Leemans et al., 2014; Sharawy and Serrya, 2020). Upon stimulation by pathogen-related molecular patterns or danger signals, a subset of NLRs forms complexes with apoptosis-related dot-like proteins containing Caspase recruitment domains to activate Caspase1, forming the inflammasome complex (Ma and Auid, 2023). The NLRP3 inflammasome holds a prominent position due to its regulation of Caspase1 activation. This event subsequently triggers the maturation of pro-IL-1β into IL-1β and pro-IL-18 into IL-18, thus fostering the release of inflammatory cytokines and orchestrating inflammatory reactions (Fu and Wu, 2023).

Notably, the NLRP3 inflammasome has been linked to a multitude of both acute and chronic renal disorders (Mulay, 2019; Liu et al., 2021). Animal studies have shown that inhibiting either TXNIP or NLRP3 can prevent acute kidney injury caused by rhabdomyolysis-induced ischemia-reperfusion injury (Yuan et al., 2017). Additionally, activation of the NLRP3 inflammasome in renal tubular epithelial cells has been linked to ischemia-reperfusion-induced acute kidney injury (Wang et al., 2022). Furthermore, a study by Akosua Vilaysane et al. demonstrated the role of NLRP3 in chronic kidney disease. NLRP3 knockout mice exhibited reduced renal tubular damage, inflammation, and fibrosis compared to wild-type mice (Vilaysane et al., 2010).

The NLRP3 inflammasome activation unfolds through two distinct steps: priming and activation. Initially, the NLRP3 gene transcription kick-started under the influence of the nuclear factor-κB (NF-κB), which leads to the expression of pro-inflammatory precursors like pro-IL-1β and pro-IL-18, along with NLRP3 (Elliott and Sutterwala, 2015). Subsequently, IL-1β and IL-18 undergo maturation and secretion, promoting inflammation and further activating the NFκB pathway. In the second step, various microbial or sterile stimuli, such as ATP released from damaged cells, trigger K^+^ efflux or other ionic changes that activate NLRP3 (Mangan et al., 2018; Fu and Wu, 2023). Zhong et al. (2016) found that NF-κB regulated the activation of the NLRP3 inflammasome and cytokine secretion through the NF-κB-P62-mitochondrial autophagy axis, while inhibiting the excessive secretion of IL-1β and the death of inflammatory macrophages, thereby avoiding long-term inflammation and tissue injury. Additionally, inhibition of the NF-κB/NLRP3 inflammatory process has been shown to improve diabetic nephropathy in mice (Yang et al., 2014). Therefore, NF-κB has been proposed as an upstream activator for NLRP3 inflammasomes. Current investigations have indicated that activation of both the NFκB pathway and the NLRP3 inflammasome is associated with inflammatory stages observed in various diseases, including acute and chronic kidney diseases (Foresto-Neto et al., 2018; Liu et al., 2019; Yin et al., 2021; Han et al., 2023).

Our study demonstrated that DQ stimulation increased ROS levels in HK-2 cells. ROS is a crucial factor known to initiate and activate NLRP3 inflammasomes. Consistently, studies by Yan Zhao et al. and Jiannan Liu et al. have demonstrated the indispensable function of ROS, particularly mitochondrial ROS, in NLRP3 inflammasome activation during various tissue injuries. These studies also emphasize the prospective therapeutic benefit of targeting ROS production to curb NLRP3 inflammasome activation (e.g., silencing the oxidoreductase responsible for mitochondrial ROS production) (Yu et al., 2017; Zhao et al., 2019; Liu et al., 2023).

While previous studies have reported NF-κB activation in nerve cell injury and diquat-induced kidney injury (Park and Koh, 2019; Cui et al., 2023), the precise function of the NLRP3 inflammasome pathway in DQ-induced renal injury remained unclear. To shed light on this, we examined the expression of pivotal NLRP3 inflammasome elements in both HK-2 cells and mouse kidney tissues. Our findings revealed increased levels of NLRP3, Caspase1 p20, IL-1β, IL-18, and p-NFκB p65. These results indicate an upregulation in NF-κB phosphorylation expression and activation of the NLRP3 inflammasome. Thus, our study confirms the participation of the NF-κB/NLRP3 inflammasome axis in the development of DQ-induced kidney injury.

## 5 Conclusion

As far as we know, this research pioneers the impact of EGCG-RBCm/NPs on the inflammasome cascade in DQ-triggered renal damage. It offers novel proof concerning the role of the NF-κB/NLRP3 inflammasome pathway in DQ-induced kidney injury and highlights the therapeutic promise of EGCG-RBCm/NPs within this context. EGCG-RBCm/NPs exhibit therapeutic potential for DQ-induced kidney injury by suppressing ROS generation and attenuating apoptosis, potentially through inhibition of the NF-κB/NLRP3 inflammasome pathway. Nevertheless, more in-depth studies are required to fully understand the underlying mechanisms of EGCG-RBCm/NPs. This study provides a strategy for utilizing EGCG-RBCm/NPs as a biomimetic nanomedicine for treating renal injury caused by diquat poisoning.

## Data Availability

The original contributions presented in the study are included in the article/Supplementary Material, further inquiries can be directed to the corresponding author.
